# Computational Design of an Electro-Organocatalyst for Conversion of CO_2_ into Formaldehyde

**DOI:** 10.1021/acs.jpca.3c07806

**Published:** 2024-02-27

**Authors:** Foroogh Khezeli, Craig Plaisance

**Affiliations:** Cain Department of Chemical Engineering, Louisiana State University, Baton Rouge, Louisiana 70803, United States

## Abstract

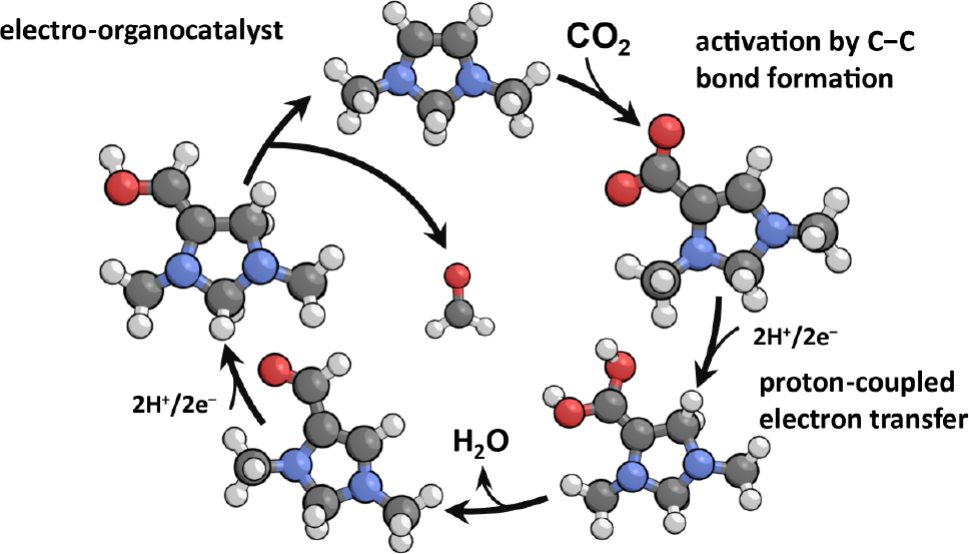

Density functional theory calculations employing a hybrid implicit/explicit solvation method were used to explore a new strategy for electrochemical conversion of CO_2_ using an electro-organocatalyst. A particular structural motif is identified that consists of an electron-rich vicinal enediamine (>N–C=C–N<) backbone, which is capable of activating CO_2_ by the formation of a C–C bond while subsequently facilitating the transfer of electrons from a chemically inert cathode to ultimately produce formaldehyde. Unlike transition metal-based electrocatalysts, the electro-organocatalyst is not constrained by scaling relations between the formation energies of activated CO_2_ and adsorbed CO, nor is it expected to be active for the competing hydrogen evolution reaction. The rate-limiting steps are found to occur during two proton-coupled electron transfer (PCET) sequences and are associated with the transfer of a proton from a proton transfer mediator to a carbon atom on the electro-organocatalyst. The difficulty of this step in the second PCET sequence necessitates an electrode potential of −0.85 V vs RHE to achieve the maximum turnover frequency. In addition, it is postulated that the electro-organocatalyst should also be capable of forming long-chain aldehydes by successively carrying out reductive aldol condensation to grow the alkyl chain one carbon at a time.

## 1 Introduction

Electrocatalytic reduction of CO_2_ is a promising means of capturing excess energy from renewable energy sources for temporary storage or use as transportation fuels and chemical feedstocks. To date, however, no practical electrochemical CO_2_ reduction technology exists, due in large part to the lack of a suitable electrocatalyst for reducing CO_2_ at the cathode. All known electrocatalysts either suffer from low Faradaic efficiency, low current densities, high overpotentials, or low selectivity to carbon products other than CO.^1^

The most utilized electrocatalyst materials for CO_2_ reduction are transition metals, such as Cu, Ag, and Au.^2−4^ Of these, Cu is the only material that produces products other than CO, even having significant selectivity to C_2_ products, such as ethylene and ethanol. However, the current densities produced by the best of these catalysts are at least an order of magnitude too low and the overpotentials too high for practical application. The origin of the low activities of transition metal electrocatalysts for CO_2_ reduction has been shown by Peterson and No̷rskov to be related to the intrinsic scaling relations between the binding energies of different catalytic intermediates on transition metal surfaces.^5^ In particular, it does not appear to be possible for a transition metal surface to bind the *COOH intermediate strong enough for the CO_2_ activation step to rapidly occur while at the same time binding CO weak enough that it does not poison the surface. The origin of these unfavorable reaction free energies lies in the linear scaling relations observed between the binding energies of *COOH and *CO on transition metal surfaces.

Biology takes quite a different approach to reducing CO_2_, utilizing not metals but the formation of a C–C bond to an organic molecule to activate CO_2_ by the RuBisCo enzyme in the Calvin cycle. In particular, CO_2_ undergoes electrophilic addition to the C=C bond in the enol form of ribulose-1,5-bisphosphate, resulting in the formation of a carboxylate group.^6^ The addition product rapidly undergoes scission into two molecules of the 3-carbon product 3-phosphoglycerate, one of which undergoes skeletal rearrangement and reduction by NADPH to reform the initial ribulose-1,5-bisphosphate, while the other exits the catalytic cycle as the final product. Thus, one can say that the biological mechanism of CO_2_ reduction is facilitated by the organocatalyst ribulose-1,5-bisphosphate.

Reproducing the biological CO_2_ reduction pathway in vitro would be extremely challenging; however, we can still derive inspiration from it toward the design of a catalytic system for electrochemical CO_2_ reduction. Specifically, we wish to design an electro-organocatalyst that can activate CO_2_ by C–C bond formation and subsequently undergo electrochemical reduction to evolve a carbon product while not being consumed itself. By using C–C bond formation to activate CO_2_, we bypass all of the difficulties and constraints associated with the scaling relations on transition metal surfaces. Additionally, there is no mechanism for evolving CO or H_2_ from an organocatalyst without involving a metal complex or surface. Therefore, these unselective pathways that occur readily on metal electrocatalysts will not occur when using an electro-organocatalyst.

Research in organocatalysis has experienced a rapid growth in the past two decades, including a Nobel Prize in 2021. Two types of organocatalyst, N-heterocyclic carbenes (NHCs)^7^ and N-heterocyclic olefins (NHOs),^8,9^ are capable of activating CO_2_ by C–C bond formation and catalyzing its insertion into propargylic alcohols, epoxides, and aziridines. This is enabled by the presence of a highly nucleophilic carbon atom in both NHCs and NHOs, which attacks the electrophilic carbon of CO_2_ to form a zwitterionic intermediate.^8,10^ Additionally, a zwitterionic indenylammonium derivative has been reported to be capable of acting as an organocatalyst to convert CO_2_ into methanol derivatives, but using an organoboron compound as the reducing agent.^11,12^ Similar to NHCs and NHOs, the zwitterionic indenylammonium organocatalyst possesses a highly nucleophilic carbon that is capable of activating CO_2_ by C–C bond formation. Although the above-mentioned organocatalysts are all capable of activating CO_2_ by C–C bond formation, they are not capable of electrochemically reducing it.

A second class of organocatalysts capable of electrochemically reducing CO_2_ consists of organic hydride donors.^13,14^ These molecules function similarly to biological NADPH, being capable of donating a hydride to the carbon atom of CO_2_ to produce formate. The hydride donor is regenerated electrochemically, making this an electro-organocatalyst. However, it does not appear possible to obtain products other than formate, likely due to the difficulty in additional hydride transfer to formate once it is formed.

In addition to organocatalysts, it is worth mentioning that metal-containing molecular catalysts can also carry out CO_2_ electroreduction,^15^ typically to CO^16^ or formate^17^ although production of oxalate^18^ has also been reported. The mechanism to form CO is similar to the initial CO_2_ activation step on metal surfaces, proceeding through a carboxylate intermediate that subsequently undergoes dehydration and CO desorption. The metal catalyst is then electrochemically regenerated to the active reduced state at an electrode. As with metal surfaces, it is likely that the strong binding of CO to metal centers leads to unfavorably scaling relationships, while competition with hydrogen evolution leads to a reduction in selectivity.

The purpose of this manuscript is to show that a molecule possessing a vicinal enediamine (>N–C=C–N<) structural motif is theoretically capable of functioning as an electro-organocatalyst for reducing CO_2_ to formaldehyde with high turnover frequencies at reasonably low overpotentials. In particular, we use density functional theory to examine a representative molecule containing this motif for such a role. First, we outline the rationale in choosing the vicinal enediamine motif based on general principles of organic chemistry. We then present the proposed catalytic cycle for such a system that is consistent with our DFT calculations and examine how the activity and energetics of the catalytic cycle are affected by electrolyte pH, electrode potential, and p*K*_a_ of the proton transfer mediator. Finally, we discuss how under appropriate conditions this electro-organocatalyst may also be capable of producing long-chain aldehydes, a far more desirable product than formaldehyde.

## 2 Rational Design of the Electro-Organocatalyst

To motivate CO_2_ activation by C–C bond formation to an organocatalyst, we first examine the thermodynamics of CO_2_ insertion into alkanes and alkenes to form carboxylic acids. We define  based on the equilibrium constant for the thermodynamic process of inserting a molecule of CO_2_ into a C–H bond,
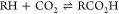


Values of  calculated from thermodynamic properties are presented in Table 1 for different R groups (at 80 °C with CO_2_ in the gas phase at 1 bar). A temperature of 80 °C was chosen since it is representative of a typical operating temperature of an electrolyzer utilizing an alkaline exchange membrane.

**Table 1:**
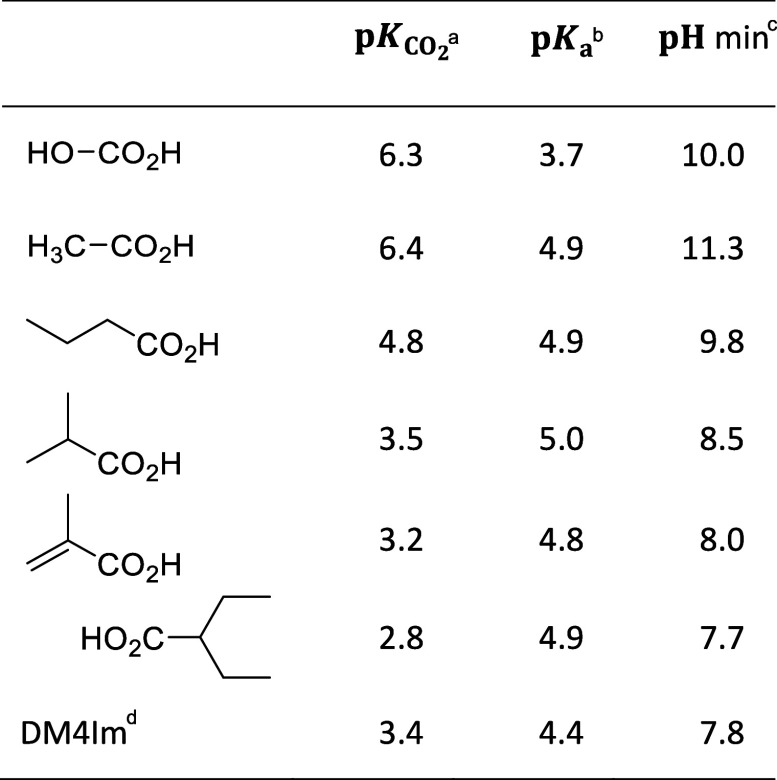
Thermodynamic Properties of CO_2_ Activation by C–C Bond Formation at 80 °C^d^

a Equilibrium constants () for CO_2_ insertion into C–H bonds.

b p*K*_a_ values for deprotonation of the resulting carboxylic acid.

c Minimum pH ( + p*K*_a_) at which the overall process is thermodynamically favorable under standard state conditions at 1 bar CO_2_.

d The last entry (DM4Im) corresponds to the DFT calculations reported in this work.

**Scheme 1:**
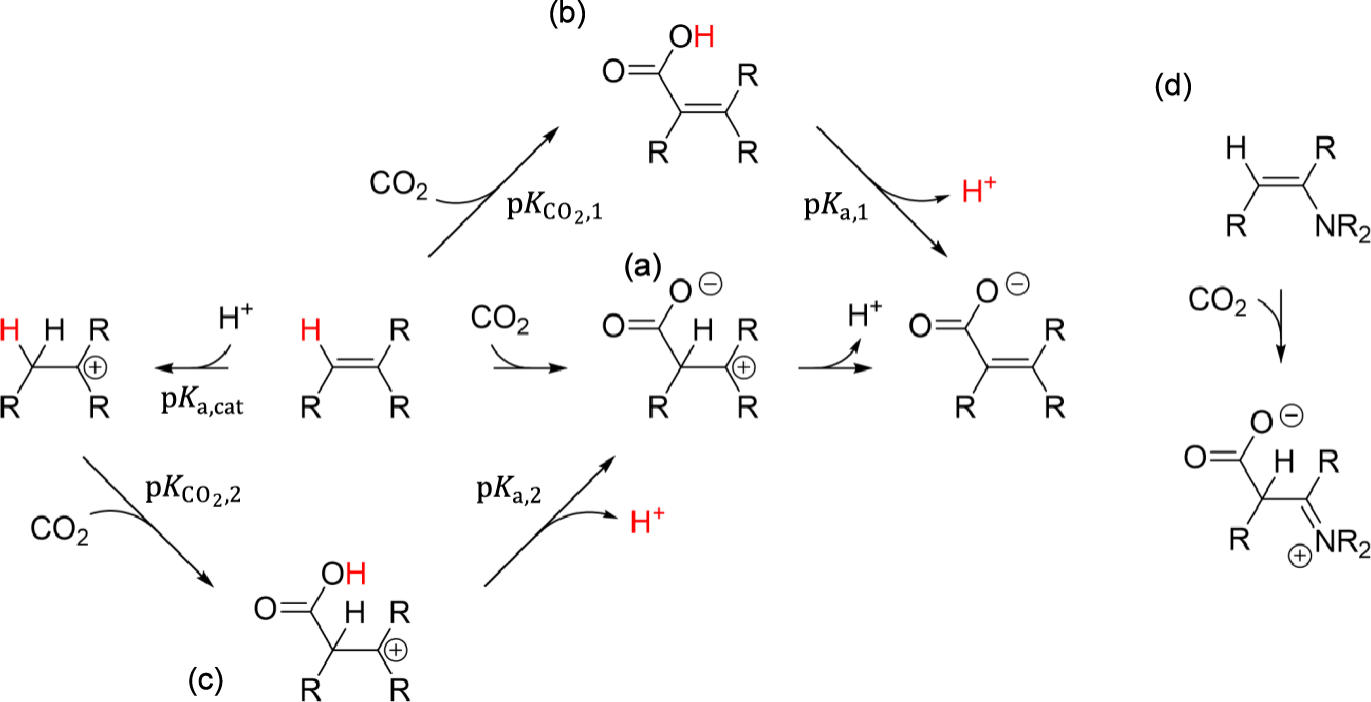
Kinetic and Thermodynamic Pathways for CO_2_ Activation by a C=C Bond Kinetic pathway for CO_2_ activation by a C=C bond proceeding through a zwitterionic intermediate. Thermodynamically equivalent Born−Haber cycle involving insertion of CO_2_ into a C−H bond followed by deprotonation of the carboxyl. Born−Haber cycle for formation of the zwitterionic intermediate. Formation of the zwitterionic intermediate from an enamine.

One can see that most of the  values (labeled  in Scheme 1b) are in the range of 2–5 and are similar to the value for CO_2_ hydration to give carbonic acid (*R* = OH). If the pH of the electrolyte is higher than the p*K*_a_ of the carboxylic acid (labeled p*K*_a,1_ in Scheme 1b), then it will deprotonate so that the overall reaction becomes,
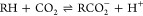


As long as the pH is higher than , this overall reaction is thermodynamically favorable. The maximum attainable pH in the presence of 1 bar of CO_2_ is limited by the cation concentration. When the cation is K^+^, the maximum concentration is 6.7 mol/L at 80 °C (corresponding to a saturated solution) resulting in a maximum pH of 9.4.^c^ It can be seen that most of these reactions are favorable at pH values lower than the maximum pH. Thus, it can be concluded that a reaction of this type is thermodynamically capable of activating CO_2_.

When considering kinetics, however, a C=C bond is required in the organocatalyst in order to activate CO_2_ by the process depicted in Scheme 1a. This involves two steps, electrophilic addition of CO_2_ to the double bond to give a zwitterionic intermediate followed by deprotonation of the C–H bond at the addition site. In order for this process to readily occur, the formation of the zwitterionic intermediate must not be too high in free energy. The formation of the zwitterionic intermediate can be rationalized in terms of the Born–Haber cycle depicted in Scheme 1c that involves protonation of the C=C bond, insertion of CO_2_ into the new C–H bond, and deprotonation of the resulting carboxylate group. Combined, the second and third steps resemble the process in Scheme 1b. It is difficult to predict whether the overall favorability of these steps () should be greater or less than the favorability of the process in Scheme 1b (). Nevertheless, we can expect the free energy to form the zwitterionic intermediate to decrease as the p*K*_a_ of the organocatalyst (p*K*_a,cat_) increases. However, its p*K*_a_ should be less than the pH so that the organocatalyst is not deactivated by protonation. This leads to the condition that an optimal organocatalyst would have a p*K*_a_ close to the maximum pH achievable under a CO_2_ atmosphere,
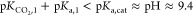
1

Note that this also encompasses the condition for thermodynamically favorable CO_2_ activation discussed in the preceding paragraph. Thus, the optimal p*K*_a_ of the organocatalyst (p*K*_a,cat_) should be around 9.

**Scheme 2:**
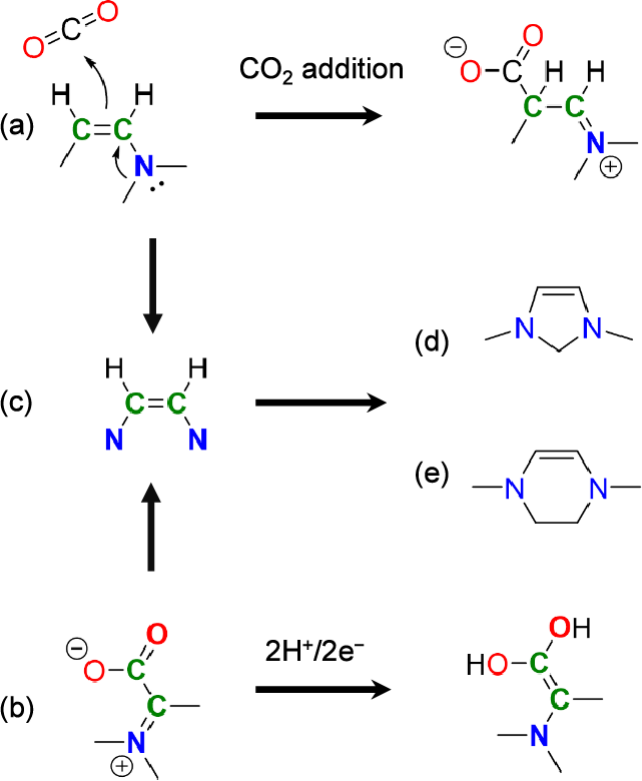
Catalytic Motifs Required for CO_2_ Reduction by an Electro-Organocatalyst Catalytic motif required for CO_2_ activation. Catalytic motif required for proton-coupled electron transfer. Combined vicinal enediamine (>N−C=C−N<) catalytic motif. 1,3-Dimethyl-4-imidazoline model catalyst used in this study. Synthetically more feasible 2,3-dihydro-1,4-dimethylpyrazine.

The simplest C=C double bond that protonates with a p*K*_a_ in this range is associated with an enamine, having typical p*K*_a_ values of 5–12 for protonation of the carbon.^20−22^ The high p*K*_a_ arises from the positive charge being concentrated on the nitrogen atom rather than a carbon atom, as depicted in Scheme 1d. Unconjugated vinyl ethers and regular alkenes are not nearly basic enough, having p*K*_a_ values close to 0^23^ and less than −12, respectively, while enolates have p*K*_a_ values around 19 that are well above the maximum pH obtainable in the presence of CO_2_. As a side note, biological CO_2_ activation by the RuBisCo enzyme occurs by electrophilic addition to an enolic C=C bond in ribulose-1,5-bisphosphate.^6^ We thus identify the N–C=C– catalytic motif as the first requirement for the electro-organocatalyst, as indicated in Scheme 2a.

Reduction of activated CO_2_ requires the transfer of electrons, thus the organocatalyst must actually be an electro-organocatalyst that is capable of readily accepting electrons from an inert cathode. This is a constraint that does not exist for the biological process since NADPH is used in the latter to accomplish reduction by hydride transfer. In order for this electron transfer to be favorable at reasonable electrode potentials, both the initial and final states of the electro-organocatalyst complex must avoid unfavorable placement of formal charge on carbon atoms. This leads us to identify the second catalytic motif (shown in Scheme 2b) consisting of a C=N double bond conjugated with the C=O bond of the carboxylate group formed during CO_2_ addition. Transfer of two electrons to this π system converts N^+^=C–C=O into N–C=C–O^–^, favorably placing the formal positive and negative charges onto nitrogen and oxygen atoms, respectively, rather than on carbon atoms.

Putting these two catalytic motifs together results in the combined motif shown in Scheme 2c which consists of a N–C=C–N (vicinal enediamine) backbone. In order to test the feasibility of such a catalytic motif, we examine the CO_2_ reduction cycle in detail on a representative molecule containing this structure, 1,3-dimethyl-4-imidazoline, shown in Scheme 2d. While this molecule is not known in the literature, it is a computationally convenient structure for studying the catalytic cycle. Other more realistic molecules having this same catalytic motif are depicted in Scheme 2e. In particular, 2,3-dihydro-1,4-dimethylpyrazine is expected to have nearly the same energetics while being synthetically more feasible. This is discussed in greater detail in Section 10.

## 3 Overall Catalytic Mechanism

**Scheme 3:**
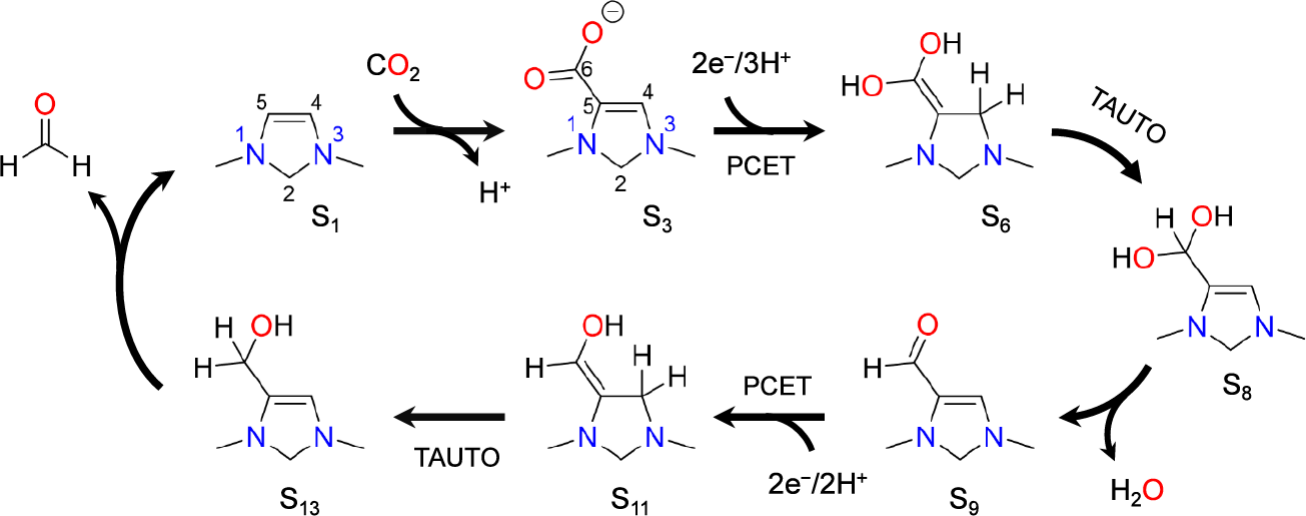
Catalytic Cycle for Reduction of CO_2_ to Formaldehyde by the 1,3-Dimethyl-4-imidazoline Model Catalyst

Having established that an electro-organocatalyst with the vicinal enediamine catalytic motif could rationally carry out CO_2_ reduction by electrophilic CO_2_ addition and electron transfer, we now turn to the mechanism by which such a catalyst would operate. The overall mechanism presented in Scheme 3 is fully justified by DFT free energy calculations on the representative molecule 1,3-dimethyl-4-imidazoline in Section 5, but we first discuss the rationale behind this mechanism before examining the energetics. The main steps are CO_2_ activation, two proton-coupled electron transfer (PCET) sequences, dehydration, and formaldehyde elimination. These main steps are connected by tautomerization steps in which protons are shuttled between different carbon atoms on the organocatalyst with the help of a proton transfer mediator (PTM) that is discussed in more detail in Section 6.

The initial state S_1_ contains six electrons in the π system, making it highly nucleophilic. This drives CO_2_ addition at the C5 position, forming a carboxylate group in S_2_. This is followed by deprotonation of C5 with the aid of the PTM to form S_3_.

State S_3_ is then protonated by the PTM at C4, isolating a N–C–CO_2_ π system in the resulting state S_4_ that readily accepts two electrons from the cathode. Proton transfer from the PTM to the oxygen atoms of the carboxylate group occurs after each of the two electron transfer steps. The two electron transfer steps result in an increase in the number of electrons in the π system from six in the initial state (S_4_) to eight in the final state (S_6_). As discussed already in Section 2, these electron transfers are particularly facile because state S_6_ formally places only one electron on each of the two carbon atoms in the N–C–CO_2_ π system, with the remaining six electrons formally existing as lone pairs on the nitrogen and oxygen atoms. As such, it avoids the unfavorable placement of formal negative charge on any carbon atom. An important point is that this is only possible once the organocatalyst has undergone CO_2_ addition, since this easily reducible motif does not exist in the initial state S_1_.

The PCET sequence is followed by a tautomerization step in which C6 (the carbon of the carboxylate group) is protonated by the PTM while C4 is subsequently deprotonated to give state S_8_. This tautomerization step has the important function of forming a C–H bond on the CO_2_-derived carbon (C6), resulting in a geminal diol. The geminal diol then undergoes dehydration to an aldehyde, leading to state S_9_.

The second PCET sequence begins with protonation of S_9_ on the carbonyl oxygen to form S_10_. The first electron transfers to the π system to form the radical species . This intermediate then undergoes proton transfer to C4 by the PTM to give . Protonation of C4 isolates the N–C–C–O pi system that is readily able to accept a second electron to yield S_11_. Somewhat analogous to the first pair of electron transfer steps, the N–C–C–O π system can accommodate six electrons without placing a negative formal charge on either carbon atom.

The second PCET sequence is again followed by a tautomerization step whereby a proton is transferred from C4 to C6 via the PTM. This is exactly analogous to the tautomerization between S_6_ and S_8_ following the first pair of electron transfer steps. The resulting intermediate S_13_ can then protonate at the C5 position via the PTM and eliminate formaldehyde to return the electro-organocatalyst to the initial state S_1_.

## 4 Density Functional Theory Calculations

We make use of a free energy profile to visualize the kinetics of the elementary steps and the overall catalytic cycle discussed in the previous section. The DFT calculations carried out to construct this profile were performed using the Vienna Ab initio Simulation Package^24^ (VASP) along with the VASPsol extension^25,26^ that allows for implicitly modeling the electrolyte using a continuum electrostatic description. The Bayesian error estimation functional with van der Waals correlation (BEEF-vdW)^27^ was used in all calculations, with further computational details given in the Supporting Information. This functional was chosen based on its balanced accuracy for describing a wide range of energetic quantities, ranging from molecular formation, reaction, and activation energies to cohesive energies of solids and chemisorption on solid surfaces. In addition, the BEEF-vdW functional includes dispersion interactions such as those that would occur between molecules and surfaces. While surfaces are not included in the present study, we envision combining this system with a solid proton transfer mediator such as a metal oxide surface in the future.

### 4.1 Hybrid Solvation Approach

The most important aspect of our simulation approach is how we handle interactions between the catalytic intermediates and the electrolyte. A purely implicit solvation method, such as the one implemented in VASPsol, does not properly account for strong hydrogen bonds that can form between the solute and solvating water molecules. To account for these interactions, we implement a hybrid implicit–explicit approach in which certain water molecules are modeled explicitly in the DFT calculations while the others are treated implicitly as a dielectric continuum. While such hybrid solvation methods have seen widespread use,^28,29^ we have encountered problems when computing free energies due to the loose vibrational modes associated with the hydrogen bonds that fall outside the validity of the harmonic approximation. To avoid this difficulty, we do not explicitly compute the vibrational contributions to the free energy arising from the hydrogen bonded water molecules and instead apply an empirical hydrogen bonding correction determined by fitting the ‘solvation’ free energy of water in itself to the experimental value. This correction, *G*_W,corr_, is found to be 0.126 eV for each explicit water participating in hydrogen bonding to the intermediate. Further details of this approach are discussed in the Supporting Information.

Free energies of intermediates and transition states were determined by adding translational, rotational, and vibrational contributions to the electronic energy *E*_0,aq_ computed by VASP with implicit solvation,
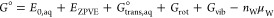
2

Translational contributions  were computed at a standard state of 1 mol/L assuming infinitely dilute ideal solution behavior. Rotational contributions *G*_rot_ were computed using the rigid rotor approximation, and vibrational contributions *G*_vib_ along with the zero-point vibrational energy *E*_ZPVE_ were computed using the harmonic approximation. All three of these contributions are computed in the absence of explicit hydrogen bonded water since these effects are already accounted for in the empirical correction *G*_W,corr_. The last term accounts for the effective chemical potential of the *n*_W_ molecules of explicitly hydrogen bonded water,
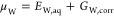
3where *E*_W,aq_ is the electronic energy of a water molecule implicitly ‘solvated’ in the electrolyte.

One issue that arises with hybrid solvation methods is how to choose the number of explicit water molecules to include. As is commonly done,^15^ we apply the ‘variational’ approach and choose the number of explicit water molecules giving the lowest free energy according to eq 2.

### 4.2 Transition States

Transition states for most steps were found by performing a roughly converged nudged elastic band calculation^30,31^ to obtain an initial guess followed by a dimer calculation^32^ to refine the transition state. Further details are given in the Supporting Information. It was found that steps involving proton transfer between the PTM and an oxygen atom ( → , S_5_ → S_6_, S_9_ → S_10_, S_14_ → S_15_, S_16_ → S_17_) did not possess a transition state, since the proton transfer occurred spontaneously upon optimization of either the initial or final state. Thus, the activation barrier for these steps is due only to the entropic cost of bringing the PTM and the electro-organocatalyst together and should be much lower than the barriers of other steps. We therefore assume that these steps are not kinetically relevant.

Transition states for electron transfer steps were found using a method that we implemented in VASP to optimize transition states of outer sphere electron transfer processes. The method is based on Marcus theory and is adapted from a method in the literature for locating conical intersections and intersystem crossings.^33^ The transition state is no longer a saddle point, but a cusp where the two potential energy hypersurfaces (with and without the added electron) intersect. The saddle point condition that the force along the reaction coordinate must vanish is replaced by the condition that the two electronic states must have the same free energy. Essentially, the free energy of the TS is being minimized subject to the constraint that the two electronic states have the same free energy. Because the two electronic states have different numbers of electrons, we must also input the electron chemical potential in order to directly compare their free energies (more precisely, their Landau potentials). Transition state energies were computing at values of the electron chemical potential ranging from −3.6 to −3.0 eV with respect to vacuum. This corresponds to −0.96 to −1.56 V vs SHE based on the absolute potential of the SHE of −4.56 V calculated with our hybrid solvation model as detailed in Section 4.5. The transition state energy was then interpolated to the value corresponding to the electrode potential. Further details of the method are given in the Supporting Information. It was found that all such energy barriers were less than 0.20 eV, indicating that activation barriers for electron transfer are mostly entropic in nature. The entropic contribution for bringing the electro-organocatalyst near the cathode is not straightforward to compute, but we assume that it is low enough that these steps are not kinetically relevant.

### 4.3 Corrections to DFT Energies

It is well-known that DFT using a semilocal exchange-correlation functional (such as BEEF-vdW) has difficulties in describing the C=O bonds in CO_2_, carboxyl groups, and to a lesser extent carbonyl groups.^34^ To reduce this error, we employ an empirical correction to the electronic energy of CO_2_ and for any molecule with a carboxylate or carbonyl group. We additionally find that a geminal diol group such as in S_7_ and S_8_ merits such a correction. These empirical corrections are determined by comparing DFT and experimental enthalpy changes for the following gas phase reactions that form CO_2_, formic acid, formaldehyde, and methanediol from methanol and water,
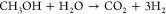

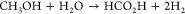

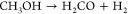

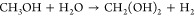


The resulting corrections are 0.52 eV for CO_2_, 0.37 eV for a carboxyl group, 0.23 eV for a carbonyl group, and 0.11 eV for a geminal diol. The carboxyl and carbonyl group corrections are also used for the corresponding enol forms of these groups. For transition states, the average value of the corrections for the initial and final states is used. Further details are given in the Supporting Information.

### 4.4 Reactant and Product Chemical Potentials

As discussed in the following section, construction of the free energy profiles requires specification of chemical potentials for the reactant and product molecules CO_2_, H_2_O, formic acid, and formaldehyde. The chemical potential of CO_2_ is computed for an ideal gas at the standard pressure *P*^◦^ of 1 bar while the chemical potential of H_2_O is computed for an ideal gas at a pressure equal to the vapor pressure *P*_sat_ of water at the reaction temperature,
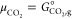
4
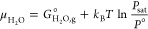
5

Note that water produced or consumed by reactions is treated differently from the explicit water molecules that only participate in hydrogen bonding. The free energies of ideal gas species are computed by a similar formula as those of the aqueous phase species, but without implicit solvation or explicit hydrogen-bonded water,
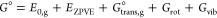
6

The translational free energy contribution is computed for an ideal gas at a standard pressure of 1 bar.

Since formaldehyde exists predominantly as methanediol at low concentrations in aqueous solution, we define the chemical potential of formaldehyde in terms of its hydration equilibrium with aqueous methanediol,
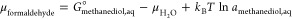
7

The activity of aqueous methanediol is set to a value corresponding to a concentration of 3.2 mmol/L. As discussed in Section 7.6, this is the maximum concentration for which elimination of formaldehyde at the end of the catalytic cycle is thermodynamically favorable. At higher concentrations, the catalytic cycle would be inhibited by methanediol/formaldehyde. However, we will discuss in Section 9 how coupling with a C–C chain growth cycle could result in low methanediol/formaldehyde concentrations while also forming multicarbon products.

The chemical potential of formic acid depends on its extent of dissociation into formate. As an approximation, we take it to be equal to the minimum of the free energies of aqueous formic acid and formate at 1 mol/L,
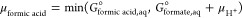
8

The free energies of aqueous methanediol, formic acid, and formate are computed using the same hybrid solvation method employed to compute free energies of the catalytic intermediates.

### 4.5 Hydrogen Atom and Proton Chemical Potentials

The electrochemical environment is accounted for by the proton and electron chemical potentials  and *μ*_*e*^–^_. These contribute a term to the free energy expression discussed in the next section which accounts for the number of protons and electrons ( and *μ*_*e*^–^_) that must be added to the reference state to form any given intermediate or transition state. We find that it is more transparent to rewrite this contribution in terms of the formal charge  and the hydrogen atom chemical potential ,
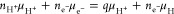
9

The advantage of this form is that the hydrogen atom chemical potential corresponds to the overall driving force for CO_2_ reduction (the electrode potential relative to the reversible hydrogen electrode), while the proton chemical potential corresponds to the electrolyte pH. The hydrogen atom chemical potential is defined using the computational hydrogen electrode approach as,^35^
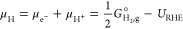
10where  corresponds to an ideal gas of H_2_ at 1 bar. The proton chemical potential is defined with respect to the equilibrium between water and hydronium,
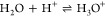


At zero pH, the hydronium activity is unity so that we can write,
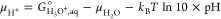
11where  is computed for a hydronium ion that is hydrogen bonded to three explicit water molecules in the Eigen configuration. The advantage of this definition for the proton chemical potential is that the experimental p*K*_a_ of hydronium (−1.7) is exactly reproduced when using this value. Using this value of the proton chemical potential we also calculate a p*K*_a_ value of 13.6 at 80 °C for deprotonating water to form hydroxide, in excellent agreement with the experimental value of 14.3. This suggests that our hybrid solvation method performs well for both hydronium-like and hydroxide-like species. Additionally, this value of the proton chemical potential gives a value of the electron chemical potential for the standard hydrogen electrode of *μ*_*e*^–^_ = – 4.56 eV which is within the experimentally measured range.

## 5 Free Energy Profile and Kinetics of the Catalytic Cycle

The free energy profile is constructed from the free energies of each catalytic intermediate () and transition state () relative to the initial state S_1_ of the catalytic cycle, all at the standard state concentration of 1 mol/L. A reactive intermediate S*_i_* formed from S_1_ by a process involving protons, electrons, and any number of other reactants and products A*_k_*,
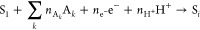
12has a relative free energy given by,
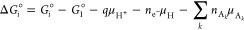
13where  and  are the absolute free energies (computed by eq 2) of S*_i_* and S_1_, respectively. The set of reactants A*_k_* consists of CO_2_, H_2_O, formic acid, and formaldehyde. The chemical potentials of the reactants and products are computed by eqs 4, 5, 7, and 8 while the hydrogen atom and proton chemical potentials are computed by eqs 10 and 11.

The relative free energy of the transition state for the reaction S_*i*_*→*S*_j_* is given by,
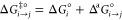
14

The activation barrier  is defined as the free energy difference between the transition state and the preceding intermediate S*_i_*. A special form is used for reactions involving proton or electron transfer, which is discussed in Section 6. For any other step converting S*_i_* to S*_j_*, possibly involving one or more reactant molecule A*_k_*, the activation barrier is given by,
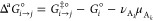
15

Where  is the absolute free energy of the transition state computed by eq 2. The stoichiometric coefficients  represent the number of each molecule A*_k_* involved in the formation of the transition state.

In addition to the standard free energies that are typically used in construction of the free energy profile, we also add a line to the plot that depicts the relative free energy corresponding to the actual concentration of each intermediate under steady-state catalytic conditions. To reduce confusion with the standard condition free energy, we will refer to this as the *resting free energy* of the catalyst—the motivation for choosing this name will become apparent later. The standard and resting free energies are related by,
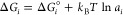
16where *a*_*i*_ is the thermodynamic activity of intermediate *i* under reaction conditions.

While the standard free energy change  for an elementary reaction step can be either positive or negative, the resting free energy change Δ^r^*G*_*i*_**_→_***_j_***=** Δ*G*_*j*_ - Δ*G*_*i*_ can only be negative, in line with the second law of thermodynamics. Based on the resting free energy change, each elementary step can be quantified as either reversible (quasi-equilibrated) if Δ^r^*G*_*i*_**_→_***_j_***≈** 0 or irreversible if Δ^r^*G*_*i*_**_→_***_j_ < 0*. One can actually identify each elementary step as reversible or irreversible based solely on the standard free energy profile. If the transition state for a step is higher in free energy than any other transition state to the right of it, then that step is irreversible; otherwise, that step is reversible. When using this rule, one must consider the cyclic nature of the catalytic cycle^36^ – the free energy profile repeats indefinitely so that a given step in one ‘iteration’ of the catalytic cycle may be rendered reversible by a higher energy transition state in the next ‘iteration.’

**Figure 1:**
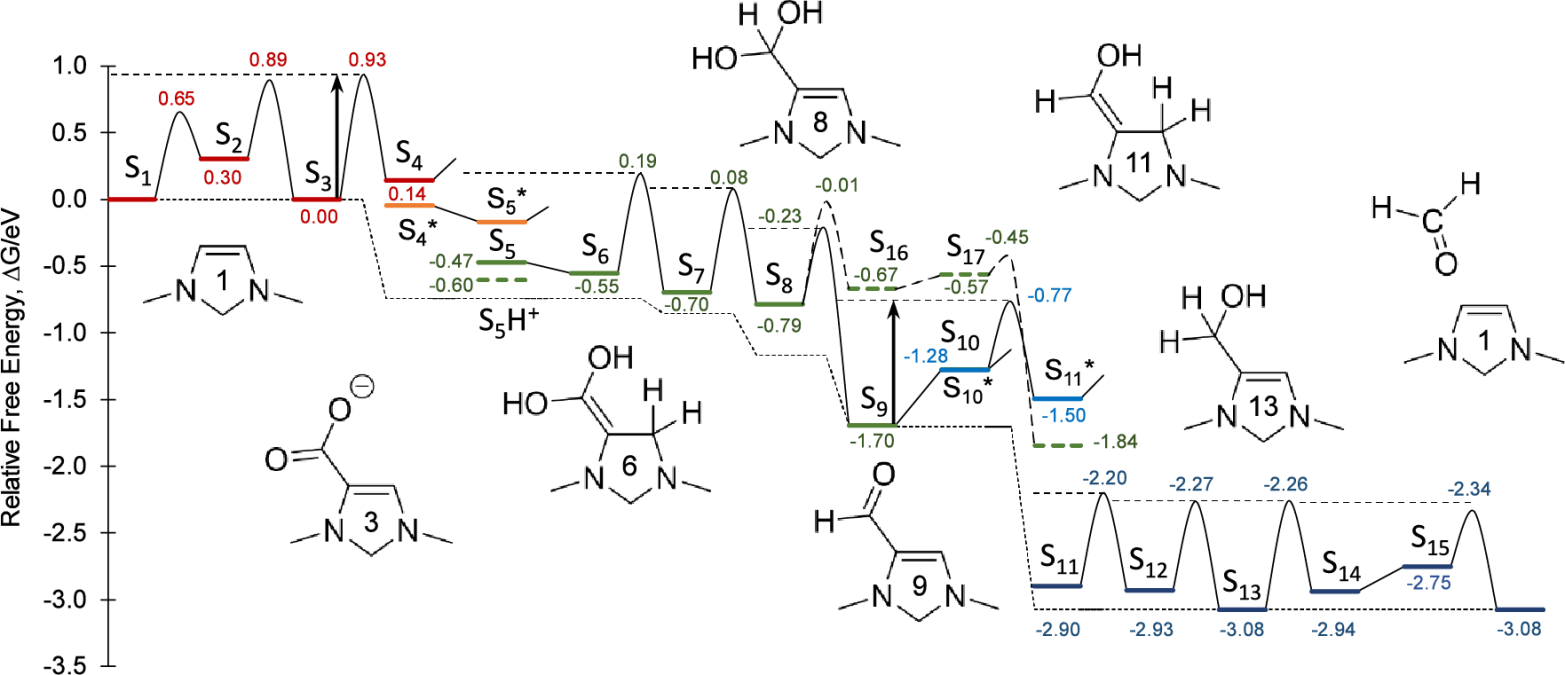
Free energy diagram of the catalytic cycle for reduction of CO_2_ to methanediol. The relative free energy of each intermediate and transition state is labled in eV. States S_16_ and S_17_ comprise a competing pathway leading to the formation of formic acid. The electron transfer steps are represented as vertical steps in the diagram (S_4_ → ,  → S_5_, S_10_ → ,  → S_11_). The upper dashed line indicates the ‘surface’ in the ‘waterfall’ analogy for interpreting the diagram, while the lower dashed line indicates the resting free energy along the reaction path. The two vertical arrows indicate the global barriers associated with the kinetically relevant steps S_3_ → S_4_ and  → . Free energies are computed at 80 °C, the catalytically optimal pH of 7.8, and the catalytically optimal potential of −0.85 V vs RHE.

A convenient mnemonic to visualize the reversibility of each elementary step is to make an analogy to water cascading down a series of barriers from left to right. If the water pools up over the transition state of an elementary step, then that step is reversible. In contrast, if the water cascades over the barrier, then that step is irreversible. The ‘surface’ of the water is depicted by the upper dashed line in Figure 1.

The ‘waterfall’ analogy can also be used to draw the resting free energy profile. This profile has the same shape as the ‘surface’ of the cascading water but is shifted down by an amount equal to the *global barrier* of the catalytic cycle that we denote Δ*G*^‡^. The global barrier is related to the turnover frequency of the catalytic cycle according to,
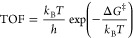
17and is determined by shifting the resting free energy profile downward until the species balance is satisfied on the total concentration of the catalyst in all possible states. In the case of an ideal solution, concentration is related to activity by *C*_*i*_ = *a*_*i*_*C*° where *C*° is the standard state concentration so that,
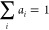
18

Due to the exponential dependence of activity on free energy, one state will typically dominate the sum and we can replace eq 16 with the approximate condition,
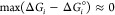
19

In other words, the resting free energy profile lies at or below the standard free energy profile. The two profiles coincide at one or more states, which we define as the *resting states* of the catalytic cycle. The typical case is for only one resting state to exist. Multiple resting states can only exist under special (typically optimal) conditions, as will be discussed in Section 8.

The technical justification for the ‘waterfall’ analogy can be seen by recognizing that every elementary step in a serial catalytic cycle must have the same net rate which is equal to the turnover frequency of the catalytic cycle. The net rate of an irreversible step is equal to its forward rate,
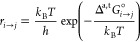
20where we define the *total barrier* as the sum of the thermodynamic barrier  and the activation barrier ,
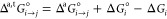
21

Thus, for all irreversible step to have the same net rate, the total barriers of all such step must be equal to the global barrier Δ*G*^‡^. Reversible steps have a forward rate faster than the turnover frequency, so that the total barrier is less than the global barrier, but the step will also proceed in the reverse direction so that the net rate is equal to the turnover frequency. One can easily see that these conditions are met when the free energy profile is constructed using the waterfall analogy.

The free energy profile depicted in Figure 1 is calculated for the catalytic cycle on the representative molecule 1,3-dimethyl-4-imidazoline at 80 °C, a pH of 7.8, and an electrode potential of −0.85 V vs RHE. This pH and electrode potential correspond to the maximum TOF for this catalyst, a topic that is discussed in detail in Section 8. Although activation barriers for proton transfer reactions were computed using formic acid as a model PTM, they are extrapolated to the optimal PTM having a p*K*_a_ value equal to the electrolyte pH as discussed in detail in the next section. At these conditions, both elementary steps involving protonation of C4 during the two PCET sequences (S_3_**→** S_4_ and **→**) are equally rate limiting. At potentials cathodic of −0.85 V vs RHE the protonation of S_3_ becomes the sole rate-limiting step, while at potentials anodic of this the protonation of  becomes the sole rate-limiting step.

## 6 Effect of Proton Transfer Mediator p*K*_a_

Many of the steps in the catalytic cycle involve proton transfer between a carbon atom on the electro-organocatalyst and a proton donor or acceptor. Water by itself is relatively poor at mediating proton transfer due to the thermodynamic unfavorability of forming hydronium or hydroxide ions. These steps consequently require the presence of a proton transfer mediator (PTM) in order to proceed at sufficient rates at moderate electrolyte pH. The PTM can be any molecule, polymer, or surface that is capable of donating and accepting protons from various catalytic intermediates in the cycle. Most biological enzymes use a PTM such as the amino group of lysine, the imidazole group of histidine, or the carboxylate groups of glutamate and aspartate to carry out acid/base reactions like keto–enol tautomerization at physiological pH.^37−40^

The PTM can exist in either a protonated state denoted HA or a deprotonated state denoted A^–^. It is assumed that these states are equilibrated under reaction conditions so that their thermodynamic activities are related to their p*K*_a_ and the pH of the electrolyte according to,
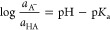
22

In the ideal solution limit where  is equal to the total concentration of the PTM, the exponential dependence of the activities on the p*K*_a_ and pH leads to the approximate expressions,
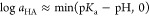
23
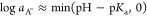


When the electrolyte pH is less than the p*K*_a_ of the PTM, the latter exists predominantly in the protonated form. As such, a protonation step such as S_3_**→** S_4_ can be written as two processes,
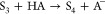

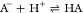


The first process involves proton transfer from the protonated PTM to S_3_ while the second process involves regeneration of the protonated PTM from the deprotonated form. Assuming that protonation of the PTM is fast and quasi-equilibrated, the effective activation barrier of the complete process is equivalent to the intrinsic kinetic barrier of the first process,
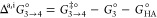
24where HA is taken to be at the standard concentration of 1 mol/L.

In the opposite case where the electrolyte pH is greater than the p*K*_a_ of the PTM, the latter exists predominantly in the deprotonated form. Under these conditions, it is more transparent to represent the protonation step as occurring by protonation of A^–^ to HA followed by proton transfer from HA to S_3_,
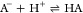

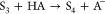


Now, the first step contributes an additional thermodynamic barrier equal to the difference in the standard and resting free energies of the protonated PTM. The effective activation barrier at any pH can thus be written approximately as,
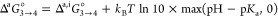
25

**Figure 2:**
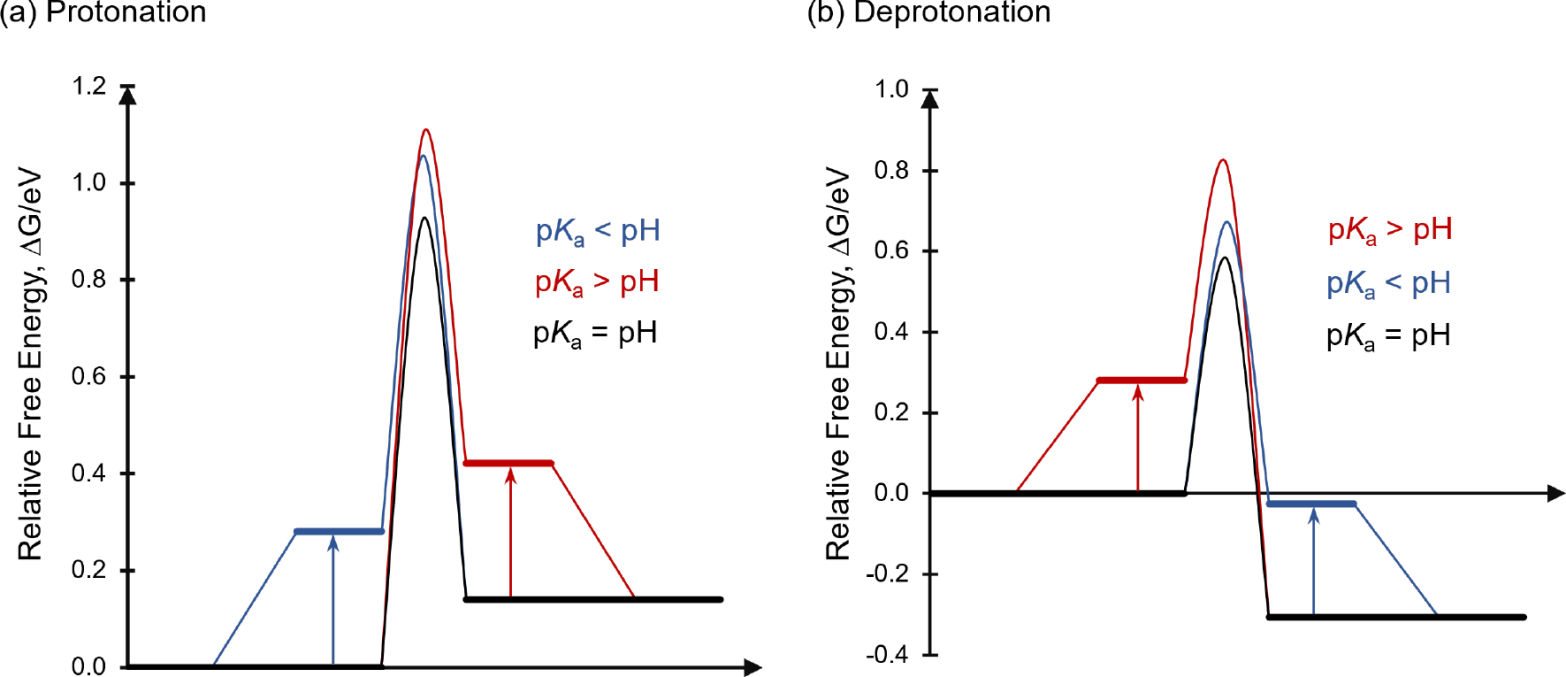
Free energy diagrams for the protonation step S_3_ → S_4_ and the deprotonation step S_2_ → S_3_ for proton transfer mediators having different p*K*_a_ values relative to the pH. The blue (red) arrow shows the additional thermodynamic barrier for protonation (deprotonation) when the PTM primarily exists in the deprotonated (protonated) state. The diagram is drawn at the catalytically optimal pH of 7.8 and a temperature of 80 °C.

The presence and absence of this additional thermodynamic barrier under different pH conditions can be seen in the free energy profile depicted in Figure 2 for S_3_**→** S_4_. The same approach is also used for the other protonation steps in the catalytic cycle S_6_**→** S_7_, **→**, S_11_**→** S_12_, S_13_**→** S_14_, and S_8_**→** S_16_.

A similar analysis can be carried out for a step deprotonation step such as S_2_**→** S_3_. When the pH is greater than the p*K*_a_ of the PTM, the deprotonation step can be represented by proton transfer from S_2_ to A^–^ followed by proton transfer to A^–^ to regenerate HA,
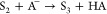

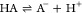


In contrast, when the pH is less than the p*K*_a_ of the PTM, the deprotonation step is represented by deprotonation of HA to A^–^ followed by proton transfer from S_2_ to A^–^,
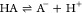

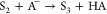


In the first case, the effective activation barrier is equivalent to the intrinsic kinetic barrier,
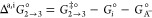
26while in the second case there is an additional thermodynamic barrier due to the difference in the standard and resting free energies of A^–^. The effective activation barrier at any pH is then approximately,
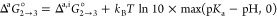
27

This approach is also used for the other deprotonation steps S_7_ → S_8_ and S_12_ → S_13_.

Examining eqs 25 and 27, it is obvious that the p*K*_a_ of the PTM will have an effect on the thermodynamic barriers that arise when the dominant state of the PTM at the electrolyte pH is different from the state required for the reaction step. In addition to this, the p*K*_a_ of the PTM will also have an effect on the intrinsic kinetic barriers. For our DFT calculations, we used formic acid as a model PTM but extrapolate to PTMs with other p*K*_a_ values using a free energy relationship. The specific form of the free energy relationship we use is based on an analogy with Marcus theory for electron transfer reactions.^41^ The general form for S_3_ → S_4_ and other protonation steps is,
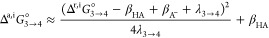
28where the reorganization energy λ_3→4_ is extracted from the intrinsic kinetic barrier explicitly calculated using formic acid as the PTM. A similar form is used for S_2_ → S_3_ and other deprotonation steps,
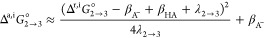
29

The intrinsic reaction free energy is defined with respect to the proton transfer step between the PTM and the intermediate. For S_3_ → S_4_ (and other protonation steps) it is defined as,
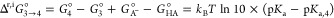
30while for S_2_ → S_3_ (and other deprotonation steps) it is defined as,
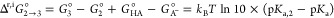
31

Here, p*K*_a,4_ and p*K*_a,2_ are the p*K*_a_ values associated with the protonation or deprotonation step, respectively. This type of free energy relationship is preferable to the more commonly used Bro̷nsted–Evans–Polanyi relationship^42^ since it has only a single parameter *λ*_*i*→*j*_ that can be determined from DFT calculations using only a single PTM. Further details of this approach are given in the Supporting Information.

The two parameters β_HA_ and  account for the free energy to form a precursor complex between the intermediate and the PTM (protonated or deprotonated form, respectively) and depend only on the identity of the PTM. These terms have two contributions, the entropic penalty to bring the PTM and the intermediate together and the penalty to break explicit hydrogen bonds between the PTM and water molecules that prevent it from approaching the intermediate. The entropic penalty is difficult to calculate so we estimate it as half the translational and rotational free energy of the model PTM we use in the calculations. The gives entopic penalties of 0.35 eV for the protonated form (formic acid) and 0.33 eV for the deprotonated form (formate). This should be an upper bound on the entropic penalty and leads to a pre-exponential factor close to 10^8^ s^–1^. The hydrogen bonding penalty is equal to the interaction between the PTM and the explicit water molecules. The protonated form (formic acid) is hydrogen bonded to one water giving a hydrogen bonding penalty of 0.06 eV, while the deprotonated form (formate) is hydrogen bonded to four water molecules giving a penalty of 0.20 eV. The total free energy penalties based on these contributions are β_HA_ 0.41 eV and  0.54 eV.

We can now discuss how the effective activation barrier of a protonation or deprotonation step will depend on the p*K*_a_ of the PTM and the pH of the electrolyte. As shown in Figure 2, both steps will exhibit a minimum effective activation barrier when the p*K*_a_ of the PTM is exactly equal to the electrolyte pH. When the PTM is more acidic, it will exist primarily in the deprotonated form and the thermodynamic barrier to convert A^–^ into HA will be equal to k_B_*T* ln 10 × (pH – p*K*_a_). At the same time, the intrinsic reaction free energy for a protonation step will decrease by this same amount and the intrinsic kinetic barrier will decrease by a fraction 0 < *α < 1* of this amount due to the free energy relationship. However, the decrease in the intrinsic kinetic barrier will always be less than the increase in the thermodynamic barrier so that the effective barrier increases overall for a protonation step. For a deprotonation step, there is no thermodynamic barrier, but the intrinsic kinetic barrier increases since the intrinsic reaction free energy is higher for a less basic PTM.

A similar argument can be made when the PTM has a p*K*_a_ greater than the electrolyte pH, leading to the conclusion that the optimal PTM has a p*K*_a_ equal to the electrolyte pH. This is in line with the Sabatier principle if one considers that the PTM is like a catalytic site that binds a proton. Since it is relatively straightforward to find a PTM having an arbitrary p*K*_a_, we will assume that the optimal PTM corresponding to the electrolyte pH is always used for proton transfer reactions.

## 7 Elementary Steps in the Catalytic Cycle

Having established the catalytic cycle and the methods used to construct the free energy profile, we now discuss the mechanistic and energetic details of the individual elementary steps involved in the cycle. While the mechanism was determined for the representative 1,3-dimethyl-4-imidazoline molecule, it is not expected to change significantly for similar molecules with the same vicinal enediamine (>N–C=C–N<) backbone.

### 7.1 CO_2_ Activation

The initial state of the organocatalyst is in equilibrium between the active state (S_1_) and an inactive state (S_0_) in which C5 is protonated.
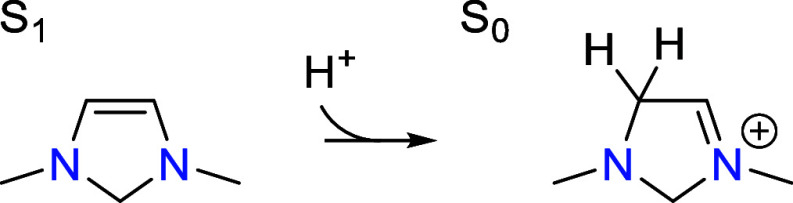


The calculated p*K*_a_ of S_0_ is 6.8 so that most of the catalyst will be deprotonated at the optimal pH of 7.8. This p*K*_a_ value is one of the key descriptors of the electro-organocatalyst, p*K*_a,cat_, as discussed in Section 2. Not only does it characterize whether the catalyst will exist in the active deprotonated state at the operating pH, but it also involves a particular quantum chemical transformation—donation of the nitrogen lone pair into the C=C double bond—that is involved in most of the other steps in the catalytic cycle. Since the p*K*_a_ value predicted by DFT is well within the range of p*K*_a_ values expected for enamines (5–12), we have high confidence in the DFT free energies computed for other steps in the cycle that also involve this transformation.

The deprotonated state activates CO_2_ by electrophilic substitution of the C5 proton in S_1_ to give S_3_. This occurs in two elementary steps, the first being electrophilic CO_2_ addition to the C=C π bond at the C5 position.
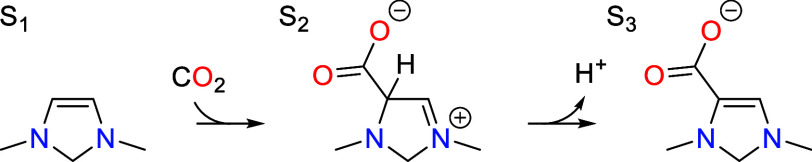


**Figure 3:**
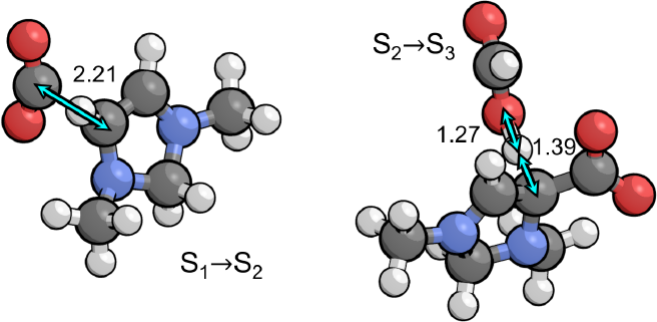
Transition states for CO_2_ addition to S_1_ and deprotonation of S_2_ by the PTM (formate) involved in CO_2_ activation. Relevant bond distances are labeled in Å.

This step is energetically favored, having a reaction energy of −0.22 eV, but is entropically disfavored due to transfer of CO_2_ from the gas phase. As a result, the reaction free energy is 0.30 eV so that S_2_ does not form in a high concentration. The driving force making addition of the unreactive CO_2_ molecule energetically favorable arises from the electron-rich π system in S_1_ containing two nitrogen lone pairs. As shown in Figure 3, the transition state involves rehybridization of the CO_2_ carbon from sp to sp^2^ and rehybridization of C5 from sp^2^ to sp^3^, having an energy barrier of only 0.13 eV. However, the entropic penalty for bringing CO_2_ from the gas phase raises the free energy barrier to 0.65 eV.

As shown in the free energy profile, the CO_2_ addition step is quasi-equilibrated due to the presence of a higher energy transition state for the subsequent deprotonation step. In this latter step, the C5 proton in S_2_ is transferred to a PTM to form S_3_. The transition state is shown in Figure 3. Since a proton is being removed, this step becomes more favorable as the pH increases. At a pH of 7.8, S_3_ has the same free energy as S_1_ so that both are resting states, which ends up determining the optimal pH of the catalytic cycle as discussed in Section 8. At this pH, the activation barrier from S_2_ → S_3_ is 0.59 eV while the total barrier is 0.89 eV with respect to the resting state S_1_. Due to the slightly higher transition state for the following step S_3_ → S_4_, the deprotonation step is close to being quasi-equilibrated. Since both steps involved in CO_2_ addition are quasi-equilibrated, the overall process is quasi-equilibrated so that S_1_, S_2_, and S_3_ have the same resting free energy.

Part of the driving force for substitution of the C5 proton by CO_2_ results from the low proton chemical potential at basic pH. At pH greater than 7.8,  is low enough to overcome the inertness of CO_2_. Interestingly, the process is nonelectrochemical so that the electrode potential has no role in driving the initial CO_2_ activation. It is also insightful to compare the free energy of S_1_ → S_3_ to an analogous thermodynamic process involving an organocatalyst without the two nitrogen atoms.
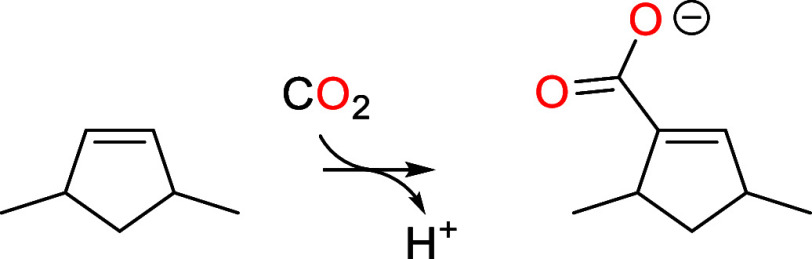


This latter process has a free energy that is 0.11 eV higher than the free energy of S_1_ → S_3_, indicating that the nitrogen atoms also play a role in thermodynamically driving CO_2_ addition. This is due to the resonance structure in which the electron-rich nitrogen lone pair delocalizes onto the carboxylate group.
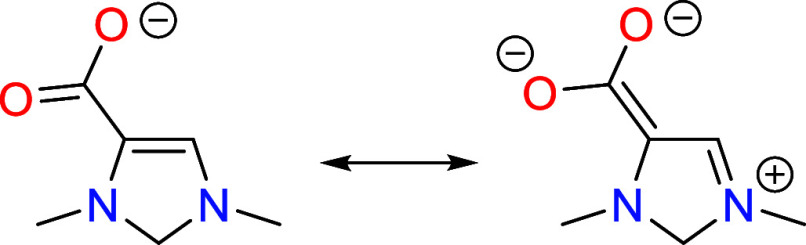


It is also interesting to compare the vicinal enediamine electro-organocatalyst to N-heterocyclic carbene (NHC) and N-heterocyclic olefin (NHO) organocatalysts. Both NHCs and NHOs are capable of activating CO_2_ by the formation of a C–C bond in a similar manner to the vicinal enediamine.^8 −10^ In fact, the vicinal enediamine is electronically similar to an NHO, both possessing an electron-rich C=C double bond that can add to an electrophile like CO_2_. Thus, the CO_2_ addition step S_1_ → S_2_ to the vicinal enediamine is analogous to CO_2_ addition to an NHO. In contrast, the nucleophilic character of NHCs is associated with an sp^2^ carbon lone pair. The main difference between NHOs and vicinal enediamines is the range of p*K*_a_ values for protonation of the nucleophilic carbon – NHOs have p*K*_a_ values ranging from 14–24 (NHCs span a similar range)^43^ while enamines have lower p*K*_a_ values in the range 5–12. As such, NHCs and NHOs would require the pH to be higher than what is possible in the presence of CO_2_ (∼10) in order to exist in the active deprotonated state.

### 7.2 First PCET Sequence

Following CO_2_ activation, S_3_ undergoes a sequence of PCET steps to yield S_6_, involving the addition of three protons from the electrolyte and two electrons from the cathode.
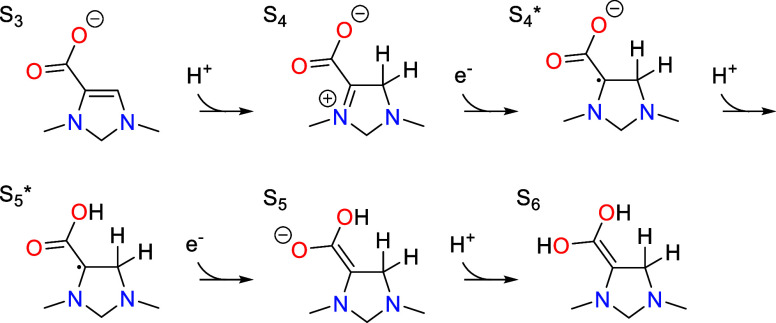


**Figure 4:**
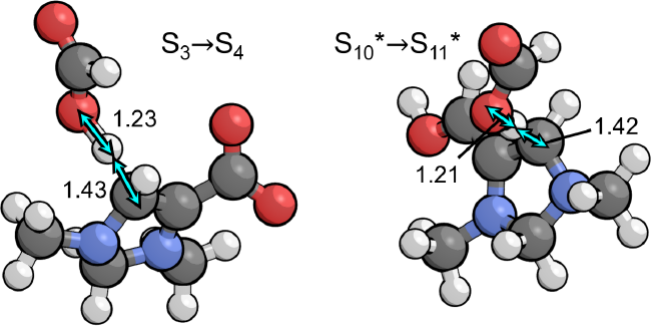
Transition states for the two kinetically relevant steps in the catalytic cycle that occur during the first and second PCET sequences, involving protonation of the C4 position by the PTM (formic acid). Relevant bond distances are labeled in Å.

This sequence of steps is initiated by proton transfer from the PTM to the C4 position on S_3_ to form S_4_, shown in Figure 4 alongside the mechanistically similar transition state for  →  of the second PCET sequence. At the optimal pH of 7.8, this step is uphill in free energy by 0.14 eV (with an associated p*K*_a_ of 5.8) and has the highest activation barrier of the catalytic cycle, 0.93 eV. Since S_3_ is the resting state for this step, there is no thermodynamic barrier so that the total barrier is equivalent to the activation barrier.

Once S_4_ is formed, it rapidly undergoes two sequential PCET steps. First, an electron is transferred from the cathode to the π system of S_4_, reducing it to the radical . This is followed by protonation of one of the carboxylate oxygen atoms to give . A second electron then transfers from the cathode to the π system of , reducing it to S_5_ which then protonates on the other carboxylate oxygen to give S_6_. The two electron transfers have standard redox potentials of −1.21 V and −1.09 V vs SHE, while the two proton transfers are associated with p*K*_a_ values of 9.5 and 9.0. The standard redox potentials of the two PCET steps are −0.54 V and −0.46 V vs RHE. Thus, all steps are thermodynamically favorable at the optimal potentials of −1.33 V vs SHE and −0.85 V vs RHE and the optimal pH of 7.8. The overall PCET sequence is highly irreversible, consuming 24% of the driving force for the full catalytic cycle.

Both electron transfers occur by an outer sphere mechanism in which they tunnel through the Helmholtz layer surrounding a chemically inert cathode into the π system of S_4_ or  in the electrolyte. As discussed already in Section 2, the outer sphere electron transfer occurs with relative ease due to the structure of the π system to which the electrons are being added. The π system in S_4_ consists of a >N^+^=C–CO_2_^–^ backbone that favorably places the positive charge arising from protonation of S_3_ onto an electron-rich nitrogen atom. The two electrons are then formally added to this nitrogen atom and the carboxylate oxygens. Both electron transfer steps are favorable at modestly low electrode potentials because all states avoid the unfavorable placement of positive or negative formal charge on any of the carbon atoms.

Rather than protonating on oxygen to give S_6_, intermediate S_5_ can also protonate on N3 to yield S_5_H^+^. This is associated with a p*K*_a_ of 9.7 and is actually lower in free energy than S_6_. It is the only case in which protonation of an intermediate on N1 or N3 is thermodynamically favorable at the optimal pH of 7.8, as seen in the Supporting Information. However, as seen in Figure 1, S_5_H^+^ is still higher in free energy than the resting free energy profile so that it is not kinetically relevant.

### 7.3 Tautomerization

Once S_6_ is formed, it undergoes tautomerization whereby a proton is transferred from C4 to C6 accompanied by migration of the double bond.
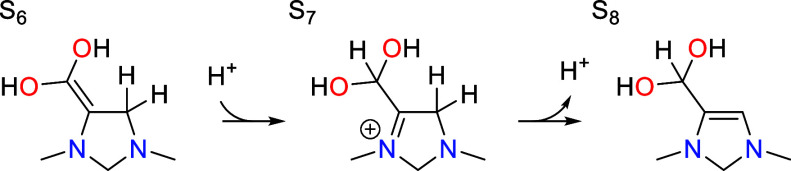


This step is formally a C=C bond migration, but occurs without forming a carbenium ion intermediate due to the presence of the nitrogen atom (N1) adjacent to C5. This allows for protonation of C6 with the formal positive charge accumulating on N1 instead of on C5, leading to an intermediate S_7_ that is far more stable than the carbenium intermediates typically associated with acid catalyzed C=C bond migration. In fact, S_7_ is even lower in free energy than S_6_ by −0.15 eV at the optimal pH of 7.8 since the step has an associated p*K*_a_ value of 9.8. Deprotonation of S_7_ at the C4 position then leads to S_8_, with a further reduction in standard free energy of −0.09 eV.

Protonation of C6 is an example of polarity reversal, or umpolung, in which the electron-rich N1 renders this carbon nucleophilic when it would normally be expected to be electrophilic. This process is a common theme in organocatalysis by NHCs, enabling carbonyl carbons to act as nucleophiles in various coupling reactions.^44^ This occurs by addition of the NHC to a carbonyl group to form a Breslow intermediate where the polarity of the carbonyl carbon is reversed from electrophilic to nucleophilic.^45^ Thus, the vicinal enediamine combines functionality of both NHCs and NHOs, functioning analogously to an NHC for the protonation step S_6_ → S_7_ while functioning analogously to an NHO for the CO_2_ addition step S_1_ → S_2_.

**Figure 5:**
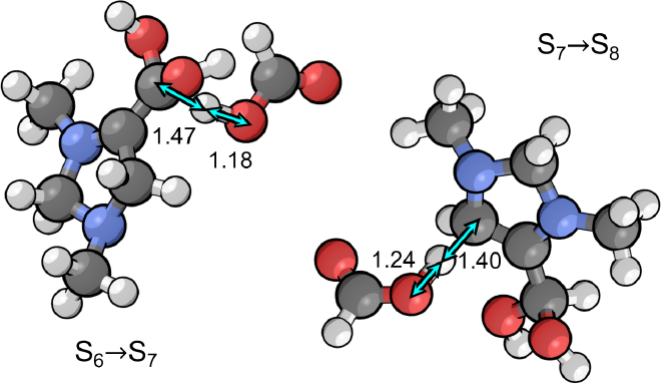
Protonation of the C6 position and deprotonation of the C4 position by the PTM (formic acid and formate, repsectively) as occur during tautomerization. Relevant bond distances are labeled in Å.

The transition states for both elementary steps involved in tautomerization are shown in Figure 5. The activation barriers for both steps are similar, being 0.75 eV for the protonation step and 0.78 eV for the deprotonation step. Neither step is rate limiting and both are irreversible. Only a small fraction of the catalyst will accumulate in S_6_ or S_7_ since the transition states for tautomerization are lower in free energy than the transition state for protonation of S_3_ that ultimately controls the rate of formation of these intermediates.

An analogous tautomerization step occurs after the second PCET sequence, converting S_11_ into S_13_.
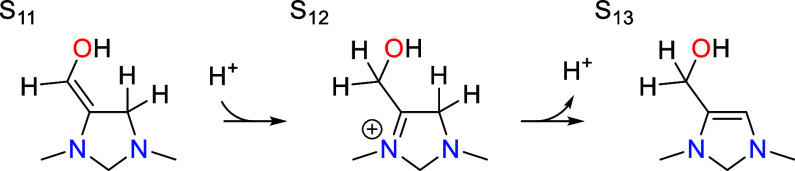


As with the first tautomerization step, the protonated intermediate S_12_ is lower in free energy than S_11_ by −0.03 eV (associated with a p*K*_a_ value of 8.2). The activation barriers for the two steps are 0.70 and 0.66 eV, slightly lower than those for the first tautomerization step.

### 7.4 Dehydration vs Formic Acid Elimination

**Figure 6:**
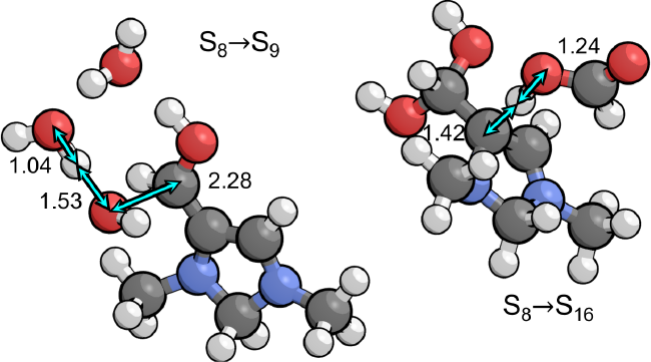
Transition states for dehydration (S_8_ → S_9_) and protonation (S_8_ → S_16_) of the geminal diol intermediate S_8_. The first evenutally leads to formaldehyde elimination while the second leads to formic acid elimination. Relevant bond distances are labeled in Å.

The geminal diol group in S_8_ readily eliminates water to give an aldehyde group in S_9_. At the optimal catalytic pH of 7.8, this is found to occur by a concerted pathway shown in Figure 6 in which one of the C–OH bonds breaks while accepting a proton that is shuttled from the other C–OH group by an additional water molecule.
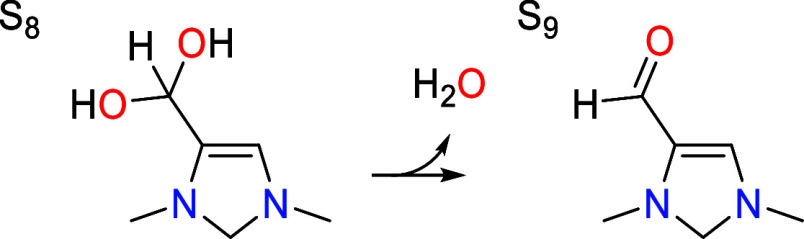


The activation barrier is 0.55 eV and the step is irreversible, consuming 31% of the driving force for the full catalytic cycle when combined with the preceding tautomerization steps.

Alternatively, S_8_ can protonate on C5 and then eliminate formic acid to return to the initial state S_1_. The mechanism proceeds by three elementary steps analogous to the formaldehyde elimination step discussed below.
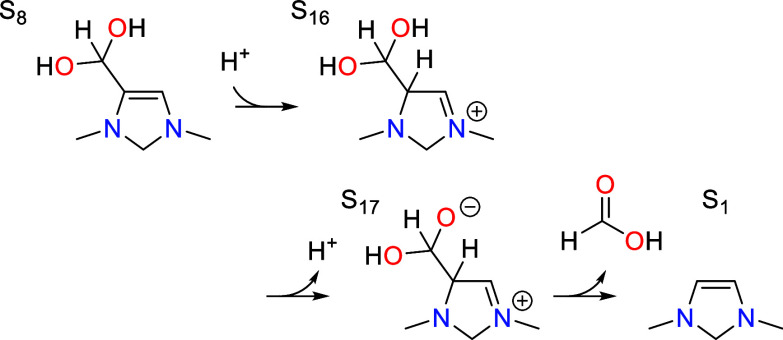


The rate-limiting protonation step has an activation barrier of 0.77 eV, significantly higher than the barrier of 0.55 eV for dehydration. Thus, formic acid production is predicted to be 3 orders of magnitude slower than formaldehyde production (which ultimately follows from dehydration).

### 7.5 Second PCET Sequence

Dehydration is followed by a second PCET sequence in which S_9_ acquires two protons from the electrolyte and two electrons from the cathode to become S_11_.
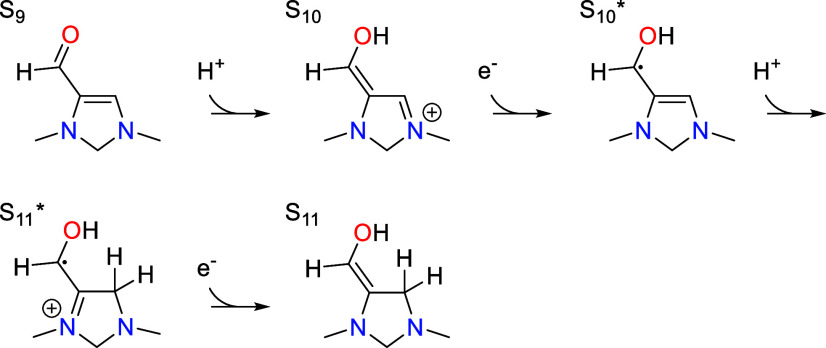


Although the overall process is similar to the first PCET sequence, the order of the protonation steps is reversed. While the first sequence commences with protonation of the C4 position, the second sequence starts with protonation of the carbonyl oxygen to give S_10_. This step has an associated p*K*_a_ value of 1.8 so that it is uphill in free energy by 0.42 eV at the optimal pH of 7.8. Protonation is followed by transfer of one electron to the π system of S_10_, converting it into the radical intermediate . The standard redox potential of the PCET step S_9_ →  is −1.27 V vs RHE so that it is uphill in free energy by 0.42 eV at the optimal potential of −0.85 vs RHE. Both of these steps are quasi-equilibrated with the resting state S_9_.

The kinetically relevant step of the second PCET sequence is protonation of  at the C4 position to yield , which was shown in Figure 4. This step has an activation barrier of 0.51 eV that combines with the thermodynamic barrier of 0.42 eV associated with S_9_ →  to give a total barrier of 0.93 eV. This is identical to the activation barrier for S_3_ → S_4_ that initiates the first PCET sequence, which is the condition that defines the optimal potential of −0.85 vs RHE. This is discussed in further detail in Section 8. The proton transfer step has an associated p*K*_a_ value of 10.9, making it downhill in free energy.

The last step of the second PCET sequence involves transfer of a second electron from the cathode to the π system of  to yield S_11_. This step is extremely favorable due to the formation of the electron deficient >N–C=C–OH^•+^ π system in the preceding step, having a standard redox potential of 0.01 V vs SHE. Consequently, this PCET sequence is highly irreversible and consumes 45% of the driving force for the full catalytic cycle, more than any other step.

### 7.6 Formaldehyde Elimination

After S_11_ tautomerizes to S_13_, the catalyst eliminates formaldehyde in the final step of the cycle to regenerate the initial state S_1_.
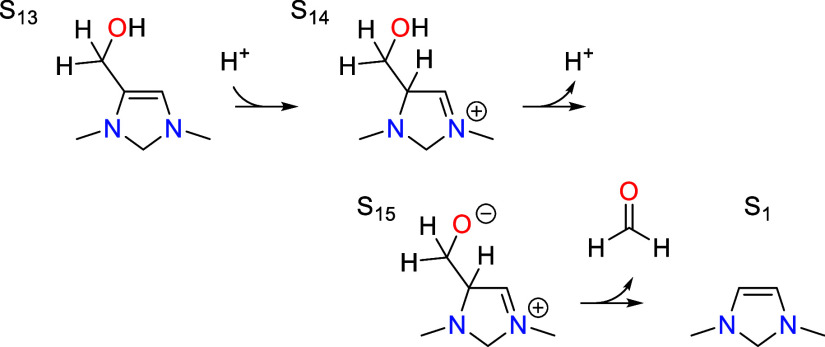


**Figure 7:**
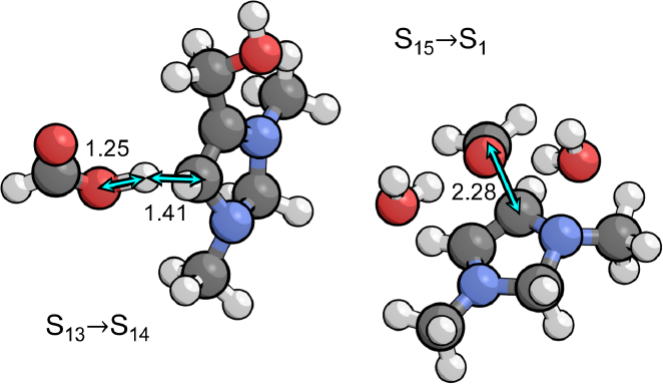
Transition states for protonation of the C5 position by the PTM (formic acid) and subsequent elimination of formaldehyde. Relevant bond distances are labeled in Å.

Formaldehyde elimination involves three elementary steps, the first being protonation of S_13_ at the C5 position to give S_14_. This is analogous to the reverse of the deprotonation step involved in CO_2_ activation; the transition state is shown in Figure 7. The activation barrier is 0.81 eV, which is equivalent to the total barrier since S_13_ is the resting state. The step is slightly uphill in free energy by 0.14 eV with an associated p*K*_a_ value of 5.8.

The second step involves deprotonation of the hydroxyl group in S_14_ to yield S_15_. This step is also uphill in free energy by 0.19 eV with an associated p*K*_a_ value of 10.4. There is no additional energetic activation barrier for this step on top of the thermodynamic barrier, only an additional entropic barrier.

Finally, the C–C bond between C5 and C6 is broken to eliminate formaldehyde. The transition state is shown in Figure 7, where it can be seen that this step involves rehybridization of both carbon atoms from sp^3^ to sp^2^ with an activation barrier of 0.48 eV. Combining this with the thermodynamic barrier for S_13_ → S_15_ gives a total barrier of 0.74 eV. This step is analogous to the reverse of the CO_2_ addition step since it involves cleavage of the C–C bond between C5 and C6.

Formaldehyde elimination is only thermodynamically favorable if the concentration of methanediol is below 3.2 mmol/L. Above this limiting concentration, S_13_ is lower in free energy than S_1_ and the catalytic cycle is inhibited by formaldehyde/methanediol. As discussed in Section 9, maintaining a low methanediol concentration is dependent on the operation of a second catalytic cycle for coupling formaldehyde into multicarbon products.

## 8 Kinetic Dependence on Potential and pH

As discussed in Section 5, the maximum TOF of 0.34 s^–1^ at 80 °C is achieved at a potential of −0.85 V vs RHE and a pH of 7.8. The TOF decreases when the electrolyte pH is higher or lower than this optimal value or when the electrode potential is anodic of −0.85 V vs RHE. This behavior can be understood by examining the free energy profile depicted in Figure 1.

At the optimal potential of −0.85 V vs RHE, both proton transfer steps to the C4 position (S_3_ → S_4_ and  → ) are equally rate limiting. The transition states for both of these steps are 0.93 eV above the preceding resting states, S_3_ and S_9_ respectively. The formation of the transition state for  →  involves a thermodynamic barrier related to transfer of a proton and electron to S_9_ to form . Decreasing the potential cathodic of −0.85 V vs RHE lowers this thermodynamic barrier, thus reducing the total barrier for  →  Consequently, this step is no longer rate limiting; however, the activation barrier for the remaining rate-limiting step S_3_ → S_4_ is unchanged so that there is no change in the TOF. In contrast, increasing the potential increases the thermodynamic barrier for S_9_ → , consequently increasing the total barrier for  →  so that it becomes the sole rate-limiting step. As the total barrier of this step increases, the TOF decreases with an associated transfer coefficient of *α* = 1 since one electron is transferred between the resting state S_9_ and the transition state.

The optimal pH is determined by the relative free energy differences between S_1_, S_3_, and S_4_. At the optimal pH of 7.8, both S_1_ and S_3_ have the same free energy so that both serve equally as resting states for the rate-limiting step S_3_ → S_4_. At constant potential vs RHE, the effect of pH on the free energy of an intermediate is proportional to its charge. Raising the pH will lower the free energies of anionic intermediates like S_3_ while raising the free energies of cationic intermediates. At pH lower than the optimal value, the anionic S_3_ will increase in free energy so that S_1_ becomes the sole resting state for S_3_ → S_4_. This will contribute an additional thermodynamic barrier associated with S_1_ → S_3_ to the total barrier for S_3_ → S_4_ so that the TOF will decrease. Likewise, decreasing the pH below the optimal value will lower the free energy of S_3_ so that it becomes the sole resting state for S_3_ → S_4_. Since S_4_ is uncharged, its free energy does not vary with pH; therefore S_3_ → S_4_ becomes thermodynamically more difficult so that its activation barrier increases and the TOF decreases.

## 9 Potential for C–C Formation and Growth of Long-Chain Aldehydes

While reduction of CO_2_ to formaldehyde is an interesting process by itself, it would be far more useful to be able to convert it to multicarbon products. Fortuitously, we have also found that the vicinal enediamine catalytic motif can additionally function as an electro-organocatalyst for reductive aldol condensation of formaldehyde to form long-chain aldehydes.^46^ We briefly summarize the results since, as discussed in previous sections, this chain growth cycle is necessary for the CO_2_ reduction cycle to function without becoming self-inhibited by the formaldehyde/methanediol product.

**Scheme 4:**
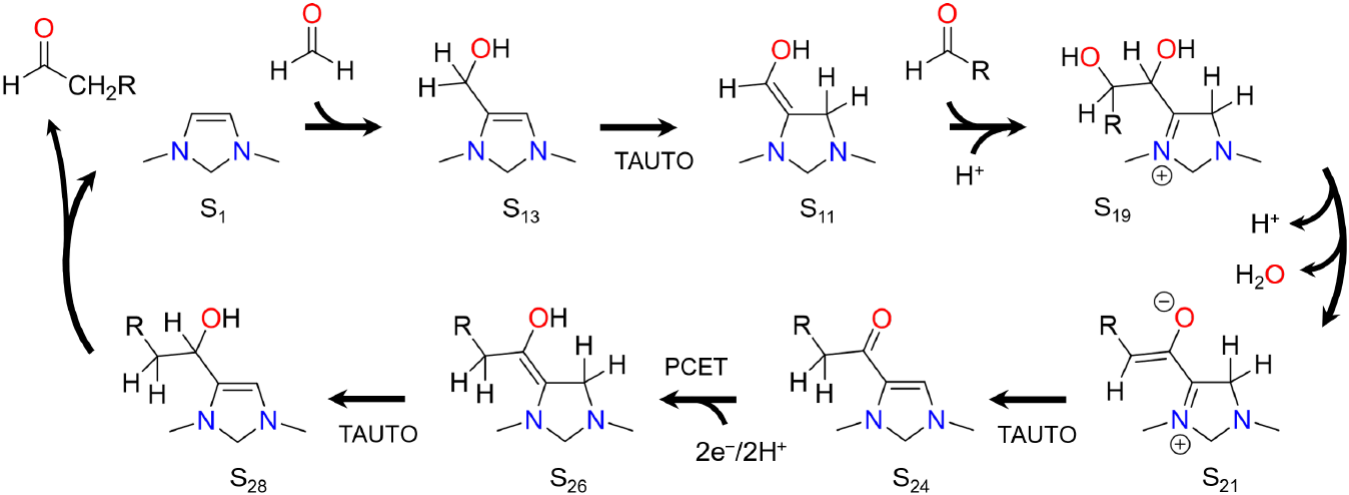
Catalytic Cycle for Aldehyde Chain Growth by Reductive Aldol Condensation with Formaldehyde. Reproduced from ref (46) Creative Commons License 2023

The mechanism found for the chain growth cycle is depicted in Scheme 4 and starts with the formation of S_13_ by the reverse of the formaldehyde elimination step. The reverse of the tautomerization step converts S_13_ into S_11_ which can then undergo aldol addition between the C6 position and another molecule of formaldehyde to form S_19_. Intermediate S_19_ then undergoes rate-limiting dehydration to S_21_ and tautomerization to S_24_ in a series of steps that are facilitated by the ability of the nitrogen atoms on the ring to accommodate formal positive charge. Intermediate S_24_ is identical to S_9_ with the hydrogen on C6 replaced by a methyl group. This intermediate can therefore undergo the same series of PCET, tautomerization, and aldehyde elimination steps as S_9_ but eliminating acetaldehyde instead of formaldehyde. Acetaldehyde itself can also undergo aldol condensation with S_11_ to eventually form propionaldehyde. Thus, each iteration of the cycle grows the starting aldehyde by one carbon unit according to,
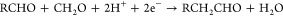


Combining this with the cycle for reduction of CO_2_ to formaldehyde gives a process for aldehyde chain growth by CO_2_,
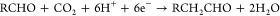


This chain growth can repeat to ultimately convert CO_2_ into arbitrarily long-chain aldehydes by the overall reaction,
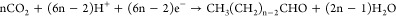
at least until the aldehyde is no longer soluble in the aqueous electrolyte.

As already mentioned, the chain growth cycle is actually necessary for the CO_2_ reduction cycle to operate. In its absence, formaldehyde addition to the electro-organocatalyst would begin to inhibit CO_2_ reduction once methanediol builds up to a concentration above the limiting concentration of 3.2 mmol/L. Thus, a rapid chain growth cycle is required to keep the methanediol concentration below this limit. Fortunately, our calculations show that the coupled CO_2_ reduction and chain growth cycles result in a methanediol concentration below this limit of 0.87 mmol/L so that major inhibition would not occur.^46^

## 10 Synthesis of an Electro-Organocatalyst with the Vicinal Enediamine Catalytic Motif

Having identified the vicinal enediamine (>N–C=C–N<) catalytic motif as possessing high activity for electrochemical reduction of CO_2_ to formaldehyde, the next step is to synthesize an electro-organocatalyst having this motif that is stable under reaction conditions. The model molecule used in our DFT calculations, 1,3-dimethyl-4-imidazoline (DM4Im), has never been synthesized. Further DFT calculations indicate that it should be at least thermodynamically possible to form this molecule by the condensation of glycoaldehyde, methylamine, and formaldehyde according to
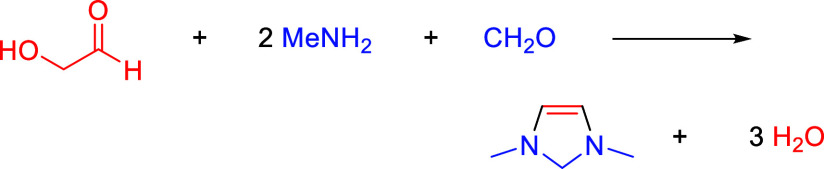


Similar condensation reactions between glycoaldehyde and amines have been reported^47^ and formaldehyde is known to form cyclic aminals by addition to diamines.^48−50^ The standard free energy of this reaction is calculated by DFT to be −0.50 eV, indicating that it is favorable thermodynamically. However, DM4Im is likely to be a powerful hydride donor that is easily capable of transferring a hydride from the C2 position to formaldehyde to reduce it to methanol.
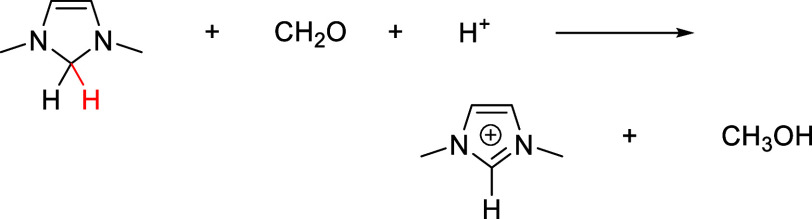


Such reactions have been reported for pyridine derivatives.^14,51^ The free energy of this hydride transfer is calculated to be −1.43 eV at the optimal catalytic pH of 7.8, indicating an extremely favorable process that is driven by restoration of aromaticity in the 1,3-dimethylimidazolium product. This makes it is unlikely that DM4Im would be a suitable electro-organocatalyst.

Replacing the hydrogens on the C2 position with methyl groups would eliminate the hydride transfer activity of DM4Im, but our DFT calculations indicate that this molecule, 1,2,2,3-tetramethyl-4-imidazoline, is unstable with respect to hydrolysis into acetone and the imine form of N,N-dimethyl-1,2-ethylenediamine (calculated reaction free energy of −0.52 eV).
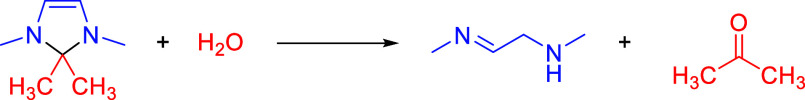


Another strategy for eliminating the hydride transfer activity is to replace the 5-membered ring with a 6-membered ring. The molecule 2,3-dihydro-1,4-dimethylpyrazine is identical to DM4Im except that the catalytically inactive methylene at the C2 position is replaced by – CH_2_–CH_2_– making it a far less active hydride donor. While no synthesis appears to exist for this molecule either, we calculate that its formation should be thermodynamically favorable from the condensation of N,N-dimethyl-1,2-ethanediamine and glycoaldehyde (calculated reaction free energy of −0.81 eV).
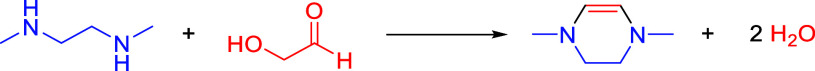


Noncyclic molecules such as N-tetramethyl-1,2-ethylenediamine could also function as the electro-organocatalyst and would follow a similar synthesis by condensation of dimethylamine and glycoaldehyde.
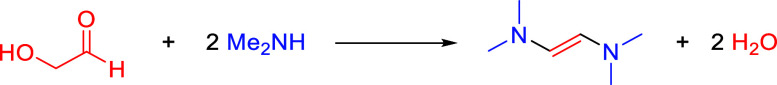


However, the reaction free energy of −0.14 eV calculated for its formation is not as favorable as for the cyclic species due to entropic effects. Therefore, more uncertainty exists as to whether this reaction is thermodynamically favorable, and a certain amount of hydrolysis is likely to occur in the reaction environment. This reaction has been reported in the literature, but in the presence of a hydrogenation catalyst that irreversibly converts the vicinal enediamine to a diamine.^47^

## 11 Conclusions

In summary, we have identified an electro-organocatalyst structure that is shown by density functional theory calculations to be active for the reduction of CO_2_ to formaldehyde. The key feature of the catalyst is a vicinal enediamine (>N–C=C–N<) catalytic motif that enables electrophilic addition of CO_2_ to the C=C bond while subsequently allowing for efficient electron transfer from a chemically inert cathode along with facile tautomerization. Activation of CO_2_ by the formation of a C–C bond avoids the constraints arising from the scaling relations associated with metal surfaces between the free energies of the *CO_2_H and *CO intermediates. Additionally, there are no feasible pathways for the competing formation of CO and H_2_ that are prevalent on transition metal electrocatalysts. Interestingly, this catalyst has similarities to biological CO_2_ activation by RuBisCo, which also occurs by electrophilic addition to a C=C bond.

Addition of CO_2_ is enabled by the electron-rich vicinal enediamine π system that results from having two nitrogen atoms adjacent to the C=C bond. This stabilizes both the direct intermediate following CO_2_ addition as well as the more stable product formed from a subsequent deprotonation step of the C5 position. After CO_2_ activation, the C4 position is protonated in one of the rate-determining steps of the catalytic cycle, resulting in a >N^+^=C–CO_2_^–^ π system that readily undergoes a sequence of two proton coupled electron transfer (PCET) steps. The electron transfers occur by an outer sphere mechanism from a chemically inert cathode, which is facilitated by the unique ability of the >N^+^=C–CO_2_^–^ π system to avoid unfavorable placement of formal charge on either of the carbon atoms; the two electron instead formally transfer to the nitrogen and oxygen atoms. The following tautomerization and dehydration steps are also facilitated by the ability of the electron-rich nitrogen atoms to accommodate formal positive charge. A second sequence of PCET and tautomerization steps then occurs that is similar to the first sequence, with protonation of the C4 position contributing the second rate-determining step of the cycle. Finally, elimination of formaldehyde occurs by a mechanism that is analogous to the reverse of the CO_2_ activation mechanism.

The catalytic cycle is found to give the highest turnover frequency of 0.34 s^–1^ at a pH of 7.8 and an electrode potential cathodic of −0.85 V vs RHE. The optimal pH is determined by the condition that the overall CO_2_ activation step results in no change in standard free energy, which leads to the minimum total barrier for the subsequent rate-limiting protonation step that initiates the first PCET sequence. The driving force for CO_2_ activation increases at higher pH, but the subsequent protonation step then becomes more difficult. The optimal potential is determined by the condition that the total barrier for the second PCET sequence be equal to the total barrier for the first PCET sequence. A more anodic potential will decrease the rate of the second PCET sequence so that it becomes the sole rate-limiting step, while a more cathodic potential will not result in any further increase in the turnover frequency since the first PCET sequence then becomes the sole rate-limiting step.

Although our conclusions are based entirely on density functional theory simulations, we stress that this work opens up an entirely new avenue for CO_2_ reduction by electro-organocatalysts. A subsequent manuscript will show that this same catalyst is capable of reductively coupling the formaldehyde product to form long-chain aldehydes under identical reaction conditions, further illustrating the powerful capabilities of such electro-organocatalysts. The next logical step is to devise a practical synthesis of an electro-organocatalyst having the >N–C=C–N< catalytic motif that is stable under reaction conditions and test it for CO_2_ activation and reduction activity. This will undoubtedly be a difficult task that will require many iterations of experiment and simulation as well as extensive device engineering once a practical catalyst is identified. Nonetheless, we are optimiztic that the new directions opened up by this initial work could eventually pave the way for a new approach to electrocatalytic conversion of CO_2_ into multicarbon products.

## Supporting Information Available

Technical details of the DFT calculations, description of the hybrid solvation method, method for finding electron transfer transition states, corrections to DFT energies, free energy relation for extrapolating proton transfer barriers, structures and hydrogen bonding free energies of intermediates and transition states, free energy contributions for intermediates and transition states (PDF)

## Footnotes

c Calculated using the Electrolyte NRTL model in Aspen Plus.
